# Gamma/Delta T Cells in the Course of Healthy Human Pregnancy: Cytotoxic Potential and the Tendency of CD8 Expression Make CD56+ γδT Cells a Unique Lymphocyte Subset

**DOI:** 10.3389/fimmu.2020.596489

**Published:** 2021-02-02

**Authors:** Jasper Nörenberg, Pál Jaksó, Alíz Barakonyi

**Affiliations:** ^1^ Department of Medical Microbiology and Immunology, Medical School, University of Pécs, Pécs, Hungary; ^2^ Department of Pathology, Medical School, University of Pécs, Pécs, Hungary; ^3^ Janos Szentagothai Research Centre, University of Pécs, Pécs, Hungary

**Keywords:** gamma/delta T cells, human pregnancy, CD4, CD8, CD56, PD-1, cytotoxicity, flow cytometry

## Abstract

To date, pregnancy is an immunological paradox. The semi-allogenic fetus must be accepted by the maternal immune system, while defense against pathogens and immune surveillance cannot be compromised. Gamma/delta T cells are believed to play an important role in this immunological puzzle. In this study, we analyzed peripheral blood CD56+ γδT cells from pregnant women (1^st^, 2^nd^, and 3^rd^ trimester) and non-pregnant women by multicolor flow cytometry. Interestingly, γδT cells represent almost half of CD3+/CD56+ cells. Among γδT cells, the CD56+ population expands in the 2^nd^ and 3^rd^ trimester. CD56+ γδT cells maintained a predominantly CD4–/CD8– or CD8+ phenotype, while CD56– γδT cells were in similar rates CD4–/CD8– or CD4+ during pregnancy. Investigation of the lysosomal degranulation marker CD107a revealed a preserved elevated rate of potentially cytotoxic CD56+ γδT cells in pregnancy, while their cytotoxic strength was reduced. Furthermore, CD56+ γδT cells continuously showed a higher prevalence of PD-1 expression. CD56+ γδT cells’ rate of PD-1 increased in the 1^st^ trimester and decreased hereafter back to normal level. We correlated the cytotoxic potential and the expression of the inhibitory immune checkpoint PD-1 and were able to demonstrate that highly cytotoxic cells within this CD56+ γδT population tend to express PD-1, which might allow the inhibition of these cells after binding its ligand in the placenta. These findings should support the understanding of the complex processes, which ensure the maintenance of pregnancy.

## Introduction

Gamma/delta T cells and their possible functions in pregnancy have been the scope of many investigations over the last decades. This T cell population has a unique physiology, as it does not underlie MHC-restriction nor requires antigen processing (1, 2). Gamma/delta T cells occur at early stages of fetal development where they are probably already capable of executing defense mechanisms against intrauterine viral infections (3). To date, the exact origin and development of γδT cells is uncertain. Although, data suggests the thymus as main source of γδT cells (4), experiments with athymic mice suggested that further development sites must exist (5). Their Vδ usage divides them into two major subsets, of which one is the Vδ2 population whereas the other is often named as non-Vδ2 population. The non-Vδ2 of adult humans consists mainly of the Vδ1 phenotype and is tissue associated. The Vδ2 phenotype is strictly associated to the usage of Vγ9 and the dominant subset in peripheral blood of adult humans (6). It was shown that the Vγ9Vδ2 subset in 1-year-old humans mostly contains non-naïve cells producing INFγ (7) and it was assumed that the dominance of the Vγ9Vδ2 subset occurs due to a pathogen-derived antigen-dependent selection process. Yet, current data suggests that pathogen-reactive effector γδT cells develop before they encounter pathogens (8, 9). Although, γδT cells are mainly double-negative for the classical T cell marker CD4 and CD8, small fractions express CD4 or CD8. As research is mainly focused on the TCR repertoire of γδT cells, the importance of these major co-receptors on γδT cells has not been investigated thoroughly.

Human γδT cells are believed to play an active role in the tolerance of paternal antigens (10–13). Nevertheless, it was not possible to describe the full picture of the immunology of pregnancy and the role of γδT cells in this complex puzzle. It can be assumed, that γδT cells have a regulatory function, which supports tolerance towards the semi-allogenic fetus on the one hand and intact defense against pathogens on the other (14). In our previous work we demonstrated the possible duality of γδT cell function in murine pregnancy: Decidual γδT cells have a strong cytotoxic potential, while expressing a tolerance promoting receptor profile (15).

As with γδT cells, the role of CD56+ cells in pregnancy, especially in human early decidua, was intensely studied. CD56 or neural cell adhesion molecule is largely known as a phenotypical marker for natural killer (NK) cells, which are often defined as CD3–/CD56+ cells. However, the expression of CD56 was also demonstrated on CD3+ αβT and γδT cells as well as on dendritic cells, monocytes and non-hematopoietic cells (16–18). CD3+/CD56+ cells were often named NKT or NKT-like cells, but with growing knowledge about the immune system and the advanced opportunities of multiparameter cytometry, the borders between cell populations became more and more vague. So far, it was not possible to demonstrate the physiological function of CD56 on any lymphoid population. However, the expression of CD56 can be correlated to the state of activation of lymphoid cells (18–20). CD56+ γδT cells show increased cytotoxicity against tumors (21) and are strong in Interferon-γ production (22). Therefore, on γδT cells, CD56 should rather be considered as a marker of activation than as a lineage marker. To date, no data about CD56-expressing γδT cells in pregnancy is available.

The basic setting of the immune system changes during pregnancy. In the first trimester, during implantation, trophoblast invasion and placentation, the maternal immune system shows a strong pro-inflammatory response (23), which is followed by a long anti-inflammatory period, where CD4+CD25+FOXP3+ regulatory T cells and CD56bright/CD16– uterine NK cells are dominant at the feto-maternal interface (24). At the end of the third trimester, due to the lower progesterone level, pro-inflammatory cytokines (IL-8, IL-1b, IL-6, TNFα) are produced (25), recruiting neutrophils and macrophages (26) secreting proteinase (27). Furthermore, the percentages of tolerance promoting regulatory T cells and decidual NK cells are decreasing. The elevated levels of IL-6 lead to high expression of oxytocin-receptor, which allows oxytocin to induce contractions and labor (28).

Here, we provide first data about CD56+ γδT cells in the course of pregnancy regarding cytotoxic potential and a possible regulation mechanism. Cytotoxicity is believed to be important in the defense against pathogens, tumor surveillance as well as the regulation of immune responses and tolerance (29–31). Those functions must be ensured during the whole time of pregnancy, while cytotoxicity must be regulated to avoid rejection of the fetus.

The B7-CD28 superfamily member programmed cell death protein 1 (PD-1) is a transmembrane receptor, which transmits co-inhibitory signal upon binding to its ligands PD-L1 or PD-L2 (32, 33). So far, the role of PD-1 and its ligands have been investigated in murine models of pregnancy. During murine pregnancy the expression of PD-1 in T lymphocytes did not change (34). However, according to the results of *Guleria et al.*, anti-PD-L1 or -L2 application to pregnant CBA/CaJ mice showed an increased fetal resorption rate and a reduction in litter size for anti-PD-L1 treatment, while no effect was observed in subject treated with anti-PD-L2 (35). These findings are further supported by the results of *D’Addio et al*, where blocking PD-L1 lead to an increase in the prevalence of Th17 cells and a decrease in the prevalence of regulatory T cells (36). In human pregnancy, both, PD-L1 and PD-L2, are expressed on all trophoblast cells (37, 38) as well as on decidual stroma cells and macrophages (39, 40). Compared to the periphery, higher rates of PD-1+ T cells have been described in the decidua. However, the prevalence of PD-1+ T cells was similar to that found in non-pregnant endometrium (40).

In this context, cytotoxicity and immune checkpoint molecule PD-1 are of particular interest on CD56+ γδT cells during pregnancy. We hypothesized that CD56+ γδT cells represent a cytotoxic subpopulation of γδT cells and assumed that PD-1—similarly as in αβT cells (32, 41)—could be a possible regulator of their cytotoxicity in the course of healthy human pregnancy. Furthermore, we anticipated that more functional aspects of γδT cells depend on their CD56-expression. Therefore, we investigated the expression of the co-receptors CD4 and CD8 to provide a first insight into CD56+ γδT cells’ characteristics.

## Materials and Methods

### Human Samples

Peripheral blood samples were obtained from healthy non-pregnant women between 18 and 40 years old (n = 17) from the Regional Blood Transfusion Service, Pecs, Hungary and in the Department of Medical Microbiology and Immunology, University of Pécs, Medical School, Hungary. Samples from different healthy pregnant women in the first [blood draw in gestational week (gw) 10–12; n = 16], second (blood draw in gw 23–27; n = 17) or third trimester (blood draw in gw 36 - 37; n = 17) were acquired in the Department of Obstetrics and Gynecology, University of Pécs, Medical School, Hungary. Our study was reviewed and approved by the local Ethics Committee (5643-PTE 2019) and informed consent was obtained from all participants. The study was adhered to the tenets of the most recent revision of the Declaration of Helsinki.

### Isolation of Peripheral Blood Mononuclear Cells

Peripheral Blood was diluted with phosphate buffered saline (PBS) and peripheral blood mononuclear cells (PBMCs) were isolated by Ficoll-Paque™ (GE Healthcare) gradient centrifugation. Hereafter, the cells were washed, resuspended in inactivated human serum (BIOWEST SAS) supplemented with 10% dimethyl sulfoxide and frozen at -80°C for later analysis.

### Fluorochrome Labeling and Flow Cytometric Analysis

Frozen PBMCs were thawed and washed twice in RPMI 1640 (Lonza) supplemented with penicillin (1 x 10^5^ U/L) (Lonza), streptomycin (0.05 g/L) (Lonza) and 10% fetal bovine serum (FCS) (Gibco^®^). The cells (10^6^ cells per tube in 100 µl RPMI 1640 with 5% FCS were incubated with fluorochrome-conjugated monoclonal antibodies (**Table 1**) for 30 min at 4°C. Afterwards, the cells were washed with PBS and resuspended with 1% paraformaldehyde in PBS and stored in the dark until flow cytometric measurement, performed with a Navios™ Ex (Beckman Coulter) and analyzed by using FlowJo™ version 10.6.1. Compensation matrices were calculated by FlowJo™ using CompBeads (BD™). All gates are based on fluorescence-minus-one controls (FMO) (gating strategy in **Supplementary Material** demonstrates data of one representative sample in the 3^rd^ trimester pregnancy).

**Table 1:** Used antibodies with conjugated fluorophore, used dilution, host species, clone, and the providing company.

Antibody	Fluorophore	Dilution	Host Species	Clone	Company
Anti-human-TCRγ/δ	FITC (Fluorescein isothiocyanate)	1:50	Mouse	B1	Sony Biotechnology Inc.
Anti-human-PD-1	PE (Phycoerythrin)	1:100	Mouse	EH12.2H7	Sony Biotechnology Inc.
Anti-human-CD4	Alexa Fluor® 700	1:100	Mouse	SK3	Sony Biotechnology Inc.
Anti-human-CD4	PE-Dazzle™594	1:50	Mouse	RPA-T4	BioLegend®
Anti-human-CD3	PE-Cy7	1:100	Mouse	SK7	Sony Biotechnology Inc.
Anti-human-CD8	APC-Cy7	1:100	Mouse	SK1	Sony Biotechnology Inc.
Anti-human-CD107a	Brilliant Violet 421™	1:200	Mouse	H4A3	Sony Biotechnology Inc.
Anti-human-CD56	Brilliant Violet 510™	1:100	Mouse	HCD56	Sony Biotechnology Inc.

### Activation and CD107a Cytotoxic Assay

To determine the cytotoxic potential of the investigated cell population we analyzed the cell surface expression of CD107a, which is an essential protein of the lysosomal membrane and becomes externalized upon degranulation of cytotoxic granules. We performed this well-established assay (42–45) as described by Andzelm et al. and previously presented in our publications (15, 46, 47): Cells were incubated for 4 h at 37°C and 5% CO_2_ with the fluorochrome-conjugated anti-CD107a antibodies in RPMI 1640, supplemented with 10% FCS, penicillin, streptomycin, ionomycin (1 µg/ml) (Sigma-Aldrich) and phorbol myristate acetate (25 ng/ml) (Sigma-Aldrich). Before labeling with the other monoclonal antibodies the cells were washed and resuspended in RPMI 1640 with 5% FCS. Finally, the cells were fixed in 1% paraformaldehyde and evaluated as described in the previous paragraph.

### Statistics

For comparison of data we performed multiple types of statistical analysis performed using GraphPad Prism 6. We use the Shapiro-Wilk test to check for gaussian distribution. For normal distributed data we used unpaired-samples-t-test to compare pregnant and non-pregnant donors. Analyses, including more than two groups were tested using Kruskal Wallis Test. To evaluate the relation of two corresponding sets of data from the same donor we used paired-samples-t-test. In cases, where normal distribution could not be assumed, we performed the Mann-Whitney test for unpaired samples and the Wilcoxon test for paired samples. Differences were determined as significant, if the *p*-value was equal to or less than 0.05. The level of significance is indicated in the text in parentheses. The respective mean ± SEM of the presented results and figures are provided in the **Supplementary Material**.

## Results

### A Small Subset of γδT Cells Shows CD56 Positivity

**Figure 1:**
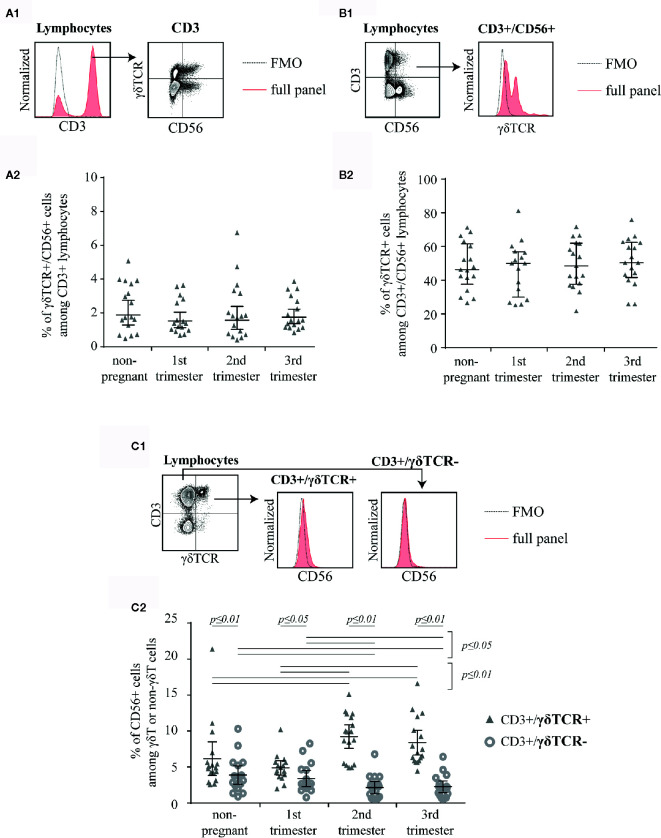
Representative, shortened gating: First gated for CD3+ cells in a histogram (the fluorescence-minus-one control (FMO) is depicted by the dotted line) then for CD56 and γδTCR **(A1)**. The mean percentage of CD56+/γδTCR+ cells among CD3+ peripheral blood lymphocytes of non-pregnant (n = 17) and pregnant [1^st^ (n = 16), 2^nd^ (n = 17), and 3^rd^ (n = 17) trimester] women is shown as horizontal line with the 95% confidence interval represented by whiskers. The individual data points are shown as triangles. Statistical analysis with Kruskal-Wallis test showed no significant alterations between the different groups **(A2)**. Representative, shortened gating: First gated for CD3+/CD56+ cells demonstrated as dot plot then for γδTCR+ cells in a histogram. The FMO is depicted by the dotted line **(B1)**. Mean percentage of γδTCR+ cells among CD3+/CD56+ peripheral blood lymphocytes of non-pregnant (n = 17) and pregnant [1^st^ (n = 16), 2^nd^ (n = 17), and 3^rd^ (n = 17) trimester] women depicted as horizontal line with the 95% confidence interval represented by whiskers. The individual data points are shown as triangles. Statistical analysis with Kruskal-Wallis test showed no significant alterations between the different groups **(B2)**. Representative, shortened gating: First gated for CD3+/γδTCR+ or CD3+/γδTCR− cells demonstrated as dot plot then for CD56+ cells in a histogram. The FMO is depicted by the dotted line **(C1)**. Mean percentage of CD56+ cells among CD3+/γδTCR+ and CD3+/γδTCR− peripheral blood lymphocytes of non-pregnant (n = 17) and pregnant [1st (n = 16), 2nd (n = 17), and 3rd (n = 17) trimester] women depicted as horizontal line with the 95% confidence interval represented by whiskers. The individual data points are shown as triangles (CD3+/γδTCR+) or circles (CD3+/γδTCR−). Statistical analysis was performed by using the Mann-Whitney test (non-pregnant vs 1st trimester vs 2nd trimester vs 3rd trimester) or the Wilcoxon test (CD3+/γδTCR+ vs CD3+/γδTCR−). Significant differences are depicted by a horizontal line above the respective data sets **(C2)**.

In this study, we aimed to investigate peripheral blood γδT cells and demonstrate their expression of CD56 in the course of human pregnancy. We found a small population of CD3+ lymphocytes, which was double-positive for γδTCR and CD56 but did not show any alterations in frequency during pregnancy or to the non-pregnant control (**Figure 1A**). However, when gating for CD3+/CD56+ lymphocytes (a typical way to characterize NKT-like cells, **Figure 1B1**), roughly half of the cells were γδTCR+ in all groups (**Figure 1B2**). Finally, we wanted to define the rate of CD56+ cells among γδT cells. Therefore, we defined γδT cells as CD3+/γδTCR+ lymphocytes and CD3+ but γδTCR− cells as non-γδT cells (**Figure 1C1**). Thereafter, we determined the prevalence of CD56+ cells within those T cell subsets. The rate of CD56+ cells was significantly higher in γδT cells compared to non-γδT cells (CD3+/γδTCR−) in all four groups. This difference was most notably in the 2^nd^ and 3^rd^ trimester (non-pregnant: *p* ≤ 0.01, 1^st^ trimester: *p* ≤ 0.05, 2^nd^ trimester: *p* ≤ 0.01, 3^rd^ trimester: *p* ≤ 0.01). Furthermore, CD56+ cells among γδT cells and non-γδT cells were rare in non-pregnant women and during the 1^st^ trimester. However, while the rate of CD56+ cells in non-γδT cells significantly decline in the 2^nd^ and 3^rd^ trimester (*p* ≤ 0.05), in γδT cells the percentage of CD56+ cells spiked in the 2^nd^ trimester (*p* ≤ 0.01). From there, the rate of CD56+ cells decreased marginally in the 3^rd^ trimester (**Figure 1C2**).

### CD56+ γδT Cells Are Predominantly Double-Negative for CD4 and CD8

**Figure 2:**
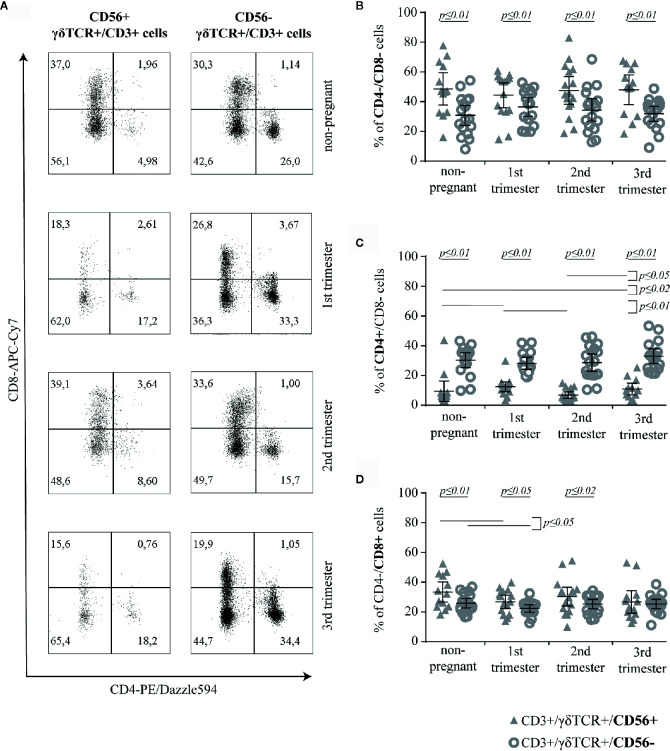
Representative CD4/CD8 dot plots of peripheral blood CD56+ or CD56− γδT cells (CD3+/γδTCR+) in non-pregnant and pregnant (1^st^, 2^nd^, and 3^rd^ trimester) women **(A)** Percentage of CD4−/CD8− **(B)**, CD4+ **(C)** and CD8+ **(D)** cells among CD56+ or CD56− γδT cells (CD3+/γδTCR+) in peripheral blood of non-pregnant (n = 17) and pregnant [1^st^ (n = 16), 2^nd^ (n = 17), and 3^rd^ (n = 17) trimester] women. The mean is depicted by a horizontal line with the 95% confidence interval represented by whiskers. The individual data points are shown as triangles (CD3+/γδTCR+/CD56+) or circles (CD3+/γδTCR+/CD56−). Significant differences between the groups were determined as followed: Panel B: Differences between the CD56+ and CD56− γδT cell subpopulation were tested by paired-samples-t-test; Panels C and D: Statistical analysis was performed by using the Mann-Whitney test (non-pregnant vs 1^st^ trimester vs 2^nd^ trimester vs 3^rd^ trimester) or the Wilcoxon test (CD3+/γδTCR+/CD56+ vs CD3+/γδTCR+/CD56−). Significant differences are depicted by a horizontal line above the respective data sets.

To determine, if the expression of CD56 on γδT cells has an impact on functional aspects of this cell population, we analyzed the expression of CD4 and CD8 on CD56+ compared to CD56− γδT cells (**Figure 2A**). Here, we found significant differences in the prevalence of double negative (CD4−/CD8−) and CD4+ cells between these two investigated subsets in all groups. The prevalence of double negative cells was permanently higher in CD56+ γδT cells (all groups: *p* ≤ 0.01) (**Figure 2B**) whereas the prevalence of CD4 was lower compared to CD56− γδT cells (all groups: *p* ≤ 0.01). However, as there was no significant alteration during pregnancy in the CD56− γδT subset, among CD56+ γδT cells, the prevalence of CD4+ cells was significantly higher in the 1^st^ and 3^rd^ trimester compared to the 2^nd^ trimester (*p* ≤ 0.01 and *p* ≤ 0.05, respectively) or in non-pregnant control group (*p* ≤ 0.01 and *p* ≤ 0.02, respectively) (**Figure 2C**). The prevalence of CD8+ cells was significantly higher among CD56+ compared to CD56− γδT cells in non-pregnant samples as well as in the 1^st^ and 2^nd^ trimester of pregnancy (*p* ≤ 0.01, *p* ≤ 0.05 and *p* ≤ 0.02, respectively). Furthermore, in both γδT cell subsets, the rate of CD8+ cells was significantly lower in the 1^st^ trimester compared to the non-pregnant control (both: *p* ≤ 0.05) (**Figure 2D**).

Further comparative analysis of the corresponding data of CD4 or CD8 positivity on CD56+ and CD56− γδT cells revealed that, in each group, the rate of CD8-expressing cells among CD56+ γδT cells was significantly higher than that of CD4+-ones (all groups: *p* ≤ 0.01; mean CD4/CD8-ratio of CD56+ γδT cells in non-pregnant, 1^st^ trimester, 2^nd^ trimester and 3^rd^ trimester group, respectively: 0.33, 0.63, 0.31, 0.58). Regarding the CD56− γδT cells we found the opposite, where in each sample group a significantly (all groups: *p* ≤ 0.01) higher rate of CD56− γδT cells were CD4+, compared with the rate of CD8+ cells among CD56− γδT cells (mean CD4/CD8-ratio of CD56− γδT cells in non-pregnant, 1^st^ trimester, 2^nd^ trimester and 3^rd^ trimester group, respectively: 1.3, 1.4, 1.28, 1.49).

### CD56+ γδT Cells Show a Strong Cytotoxic Potential

**Figure 3:**
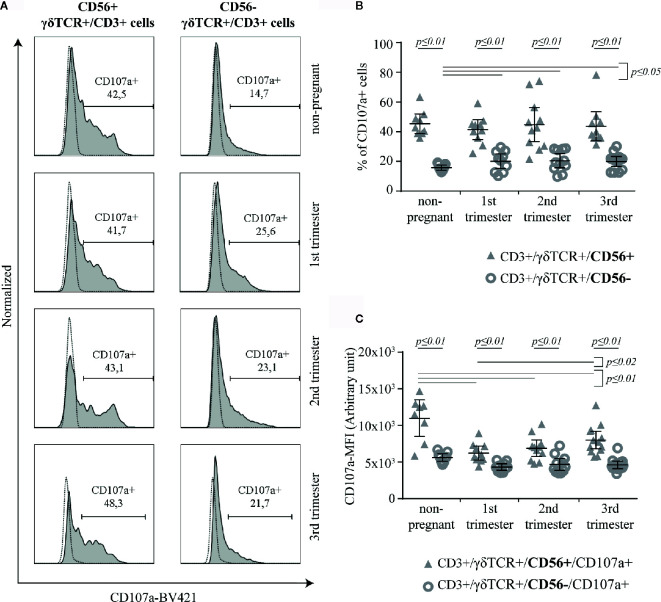
Representative CD107a histograms (the FMO is depicted by the dotted line) of peripheral blood CD56+ or CD56− γδT cells (CD3+/γδTCR+) in non-pregnant and pregnant (1^st^, 2^nd^, and 3^rd^ trimester) women **(A)** Percentage **(B)** and Mean of Fluorescence Intensity (MFI) **(C)** of CD107a+ cells within peripheral blood CD56+ or CD56− γδT cells (CD3+/γδTCR+) in non-pregnant (n = 9) and pregnant [1^st^ (n = 10), 2^nd^ (n = 10), and 3^rd^ (n = 13) trimester] women. The mean is depicted by a horizontal line with the 95% confidence interval represented by whiskers. The individual data points are shown as triangles (CD3+/γδTCR+/CD56+ or CD3+/γδTCR+/CD56+/CD107a+, respectively) or circles (CD3+/γδTCR+/CD56− or CD3+/γδTCR+/CD56−/CD107a+, respectively). Statistical analysis was performed by using the Mann-Whitney test (non-pregnant vs 1^st^ trimester vs 2^nd^ trimester vs 3^rd^ trimester) or the Wilcoxon test (CD3+/γδTCR+/CD56+ vs CD3+/γδTCR+/CD56−). Significant differences are depicted by a horizontal line above the respective data sets.

The potential for cytotoxic activity of γδT cells was determined by the surface expression of CD107a upon activation (**Figure 3A**). Although, the rate of CD107a+ cells was significantly higher among CD56+ γδT cells compared to the CD56− subset in all groups (all groups: *p* ≤ 0.01), this rate did not alter considerably during pregnancy. However, the percentage of CD107a+ cells among CD56− γδT cells was significantly higher in pregnancy compared to the non-pregnant control (*p* ≤ 0.05) (**Figure 3B**). For a better classification of the cytotoxic potential we determined the CD107a-mean fluorescens intensity (MFI) of CD107a+ cells in both γδT subsets. Here, the MFI was significantly higher in the CD56+ γδT subpopulation in all sampled groups (all groups: *p* ≤ 0.01). In CD56+ γδT cells, the CD107a-MFI was significantly lower during pregnancy compared to the non-pregnant control (all pregnant groups: *p* ≤ 0.01). In pregnancy, the lowest CD107a-MFI was found in the 1^st^ trimester, from there it increased significantly to the 3^rd^ trimester (*p* ≤ 0.02). The MFI of CD56− γδT cells did not alter (**Figure 3C**).

### The Rate of PD-1+ Cells Is Higher in CD56+ γδT Cells

**Figure 4:**
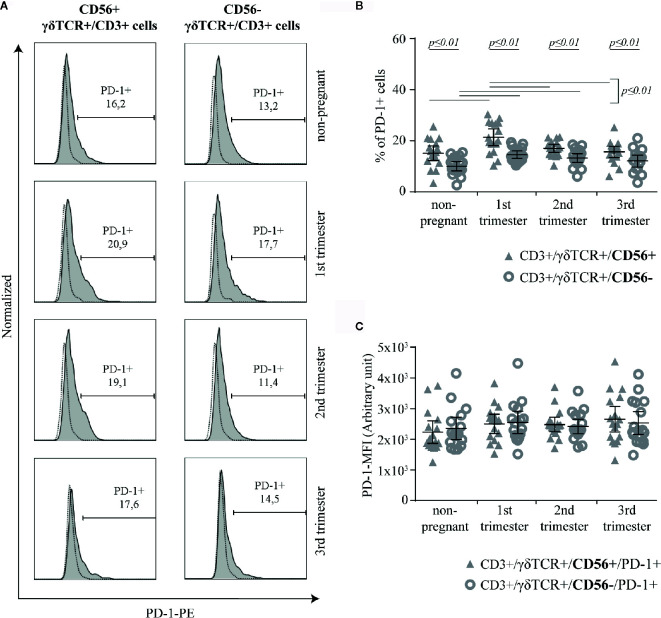
Representative PD-1 histograms (the FMO is depicted by the dotted line) of peripheral blood CD56+ or CD56− γδT cells (CD3+/γδTCR+) in non-pregnant and pregnant (1^st^, 2^nd^, and 3^rd^ trimester) women **(A)** Percentage **(B)** and PD-1-MFI **(C)** of PD-1+ cells among peripheral blood CD56+ or CD56− γδT cells (CD3+/γδTCR+) in non-pregnant (n = 17) and pregnant [1^st^ (n = 16), 2^nd^ (n = 17), and 3^rd^ (n = 17) trimester] women. The mean is depicted by a horizontal line with the 95% confidence interval represented by whiskers. The individual data points are shown as triangles (CD3+/γδTCR+/CD56+ or CD3+/γδTCR+/CD56+/PD-1+, respectively) or circles (CD3+/γδTCR+/CD56− or CD3+/γδTCR+/CD56−/PD-1+, respectively). Statistical analysis was performed by using the paired-samples-t-test to compare data between the CD56+ and CD56− γδT cell population and the unpaired-samples-t-test to compare within the same population but at different timepoints. Significant differences are depicted by a horizontal line above the respective data sets.

The surface expression of PD-1 was measured on CD56+ or CD56− γδT cells (**Figure 4A**). Compared to CD56− γδT cells, PD1+ cells were significantly more common in CD56+ γδT cells at all measured timepoints (all groups: *p* ≤ 0.01). Within the CD56+ γδT subset, the prevalence of PD-1+ cells increased significantly (*p* ≤ 0.01) in the first trimester and fell back to non-pregnant-level in the 2^nd^ and 3^rd^ trimester (both: *p* ≤ 0.01). Among CD56− γδT cells, the prevalence of PD-1+ cells was significantly higher in the 1^st^ and 2^nd^ trimester compared to the non-pregnant control (both: *p* ≤ 0.01) (**Figure 4B**). No significant difference was detected regarding the MFI of PD-1 (**Figure 4C**).

### The Co-Expression of PD-1 and CD107a on γδT Cells Correlates With Their CD56 Expression

**Figure 5:**
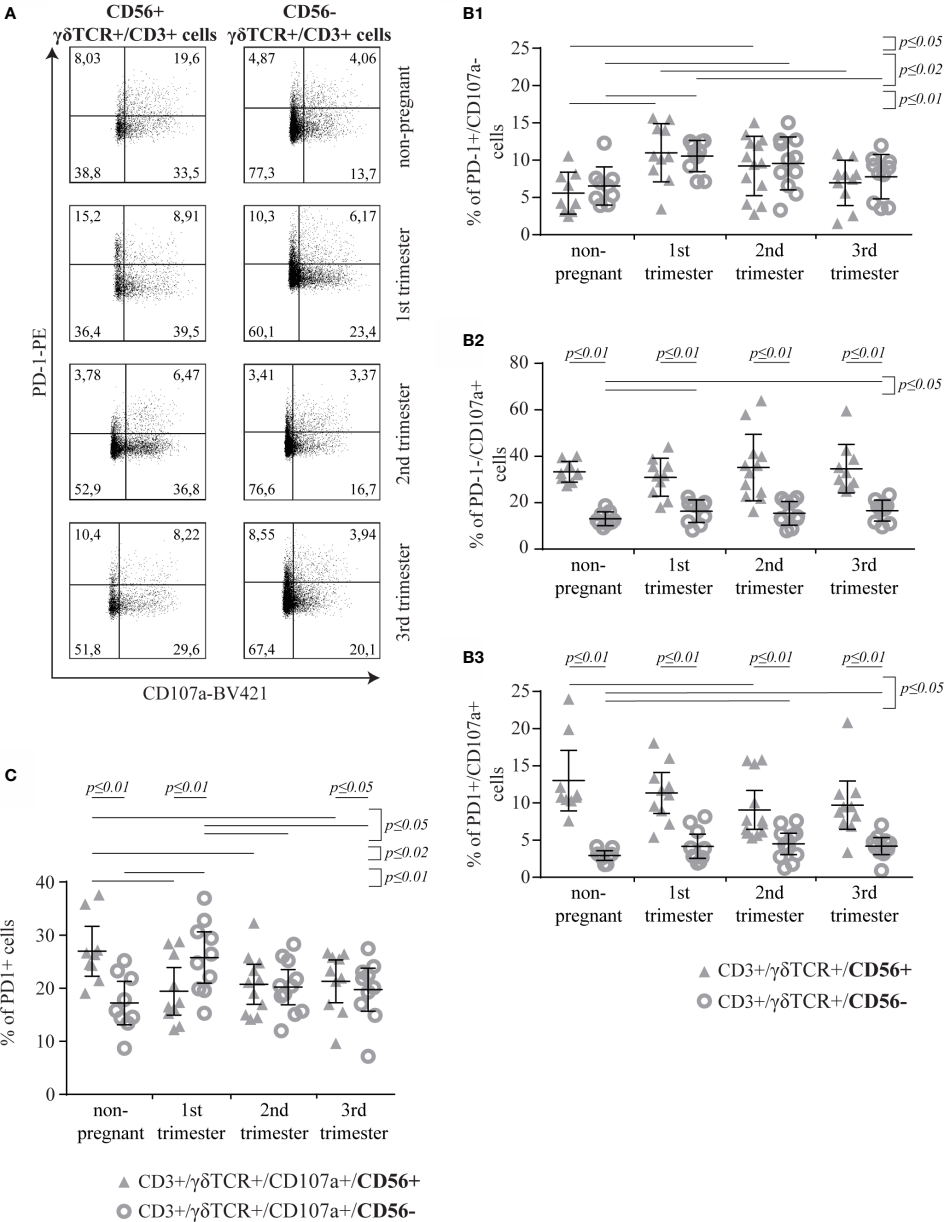
Representative PD-1/CD107a dot plots of peripheral blood CD56+ or CD56− γδT cells (CD3+/γδTCR+) in non-pregnant and pregnant (1^st^, 2^nd^, and 3^rd^ trimester) women **(A)**. Rates of PD-1+/CD107a− **(B1)**, PD-1−/CD107a+ **(B2)** and PD-1+/CD107a+ **(B3)** cells among peripheral blood CD56+ or CD56− γδT cells (CD3+/γδTCR+) in non-pregnant (n = 9) and pregnant [1^st^ (n = 10), 2^nd^ (n = 10), and 3^rd^ (n = 13) trimester] women **(B)**. Percentage of PD-1+ cells among CD107a+ cells in the peripheral blood CD56+ or CD56− γδT cells (CD3+/γδTCR+) in non-pregnant (n = 9) and pregnant [1^st^ (n = 10), 2^nd^ (n = 10), and 3^rd^ (n = 13) trimester] women **(C)**. The means of the data presented in Panels B and C are depicted as a horizontal line with the 95% confidence interval represented by whiskers. The individual data points are shown as triangles (CD3+/γδTCR+/CD56+ or CD3+/γδTCR+/CD107a+/CD56+, respectively) or circles (CD3+/γδTCR+/CD56− or CD3+/γδTCR+/CD107a+/CD56−, respectively). Statistical analysis was performed by using the paired-samples-t-test to compare data between the CD56+ and CD56− γδT cell population and the unpaired-samples-t-test to compare within the same population but at different timepoints. Significant differences are depicted by a horizontal line above the respective data sets.

Here, we aimed to investigate the co-expression of PD-1 and CD107a in CD56+ or CD56− γδT cells (**Figure 5A**). No significant difference was observed in the prevalence of PD-1+/CD107a− cells between CD56+ and CD56− γδT subsets in any study group. Within both of these subsets, the prevalence of PD-1+/CD107a− cells was significantly higher in the 1^st^ and 2^nd^ trimester than in the non-pregnant group (CD56+: *p* ≤ 0.01 and *p* ≤ 0.05, respectively; CD56−: *p* ≤ 0.01 and *p* ≤ 0.02, respectively). Furthermore, the highest rates were in the 1^st^ trimester, which were also significantly higher compared to the 3^rd^ trimester (both subsets: *p* ≤ 0.02) (**Figure 5B1**). The prevalence of PD-1−/CD107a+ cells was significantly higher among CD56+ than in CD56− γδT cells in all groups (all groups: *p* ≤ 0.01). Among the CD56− γδT cells the prevalence of PD-1−/CD107a+ cells was higher in the 1^st^ and 3^rd^ trimester of pregnancy compared to the non-pregnant control (both: *p* ≤ 0.05) (**Figure 5B2**). Compared to the CD56− subset, the prevalence of PD-1+/CD107a+ cells was significantly higher in the CD56+ γδT subset at all timepoints (all groups: *p* ≤ 0.01). Within this subset, the prevalence of double-positive cells was significantly lower in the 2^nd^ trimester compared to the non-pregnant group (*p* ≤ 0.05). In opposite, among the CD56− γδT cells, the rate of double-positive cells was the lowest in the non-pregnant group and significantly higher in the 2^nd^ and 3^rd^ trimester (both: *p* ≤ 0.05) (**Figure 5B3**).

Further comparative analysis of the corresponding data of PD-1+/CD107a+ and PD-1−/CD107a+ cells in the CD56+ and CD56− γδT subsets revealed that in both subsets and in all groups γδT cells were rather PD-1−/CD107a+ than PD-1+/CD107a+ (all *p* ≤ 0.01)

We also calculated the ratio (R) of double- and single-positive cells:


$$R=\frac{PD-1+/CD107a+}{PD-1-/CD107a+}$$

When comparing the “R” values of CD56+ and CD56− γδT subsets (mean R of CD56+ γδT cells in non-pregnant, 1^st^ trimester, 2^nd^ trimester and 3^rd^ trimester group, respectively: 0.38, 0.37, 0.26, 0.27; mean R of CD56− γδT cells in non-pregnant, 1^st^ trimester, 2^nd^ trimester and 3^rd^ trimester group, respectively: 0.22, 0.24, 0.26, 0.24) we found that the “R” value was significantly higher in the CD56+ γδT subset in all groups, except in the 2^nd^ trimester, where it was equal to the CD56− γδT subset (non-pregnant, 1^st^ trimester: *p* ≤ 0.01, 3^rd^ trimester: *p* ≤ 0.02). In the CD56+ γδT subset, this ratio was significantly lower during the 2^nd^ and 3^rd^ trimester compared to the 1^st^ trimester (*p* ≤ 0.02, and *p* ≤ 0.05, respectively) and the non-pregnant control (*p* ≤ 0.01 and *p* ≤ 0.05, respectively). In contrary, in the CD56− γδT subset, no significant alteration between the different timepoints was found.

For a better understanding of the impact of PD-1 within the cytotoxic γδT cells, we studied the expression of PD-1 on CD56+/CD107a+ and CD56−/CD107a+ γδT cells. Here, compared to CD56− γδT subset, significantly higher rate of cytotoxic CD56+ γδT cells express PD-1 in non-pregnant group (*p* ≤ 0.01) and in the 3^rd^ trimester pregnancy (*p* ≤ 0.05), whereas in the 1^st^ trimester we found the opposite result (*p* ≤ 0.01). The prevalence of PD-1+ cells in the cytotoxic CD56+ γδT subset was significantly lower in pregnancy than in non-pregnant state (1^st^ trimester: *p* ≤ 0.01, 2^nd^ trimester: *p* ≤ 0.02, 3^rd^ trimester: *p* ≤ 0.05). However, during pregnancy the rate of PD-1+ cells among the cytotoxic CD56+ γδT cell subset did not alter. Interestingly and in opposite to the cytotoxic CD56+ γδT subset, the cytotoxic CD56− γδT subset showed a significant increase of PD-1+ cells in the 1^st^ trimester (non-pregnant: *p* ≤ 0.01, 2^nd^ and 3^rd^ trimester: *p* ≤ 0.05) (**Figure 5C**).

**Figure 6:**
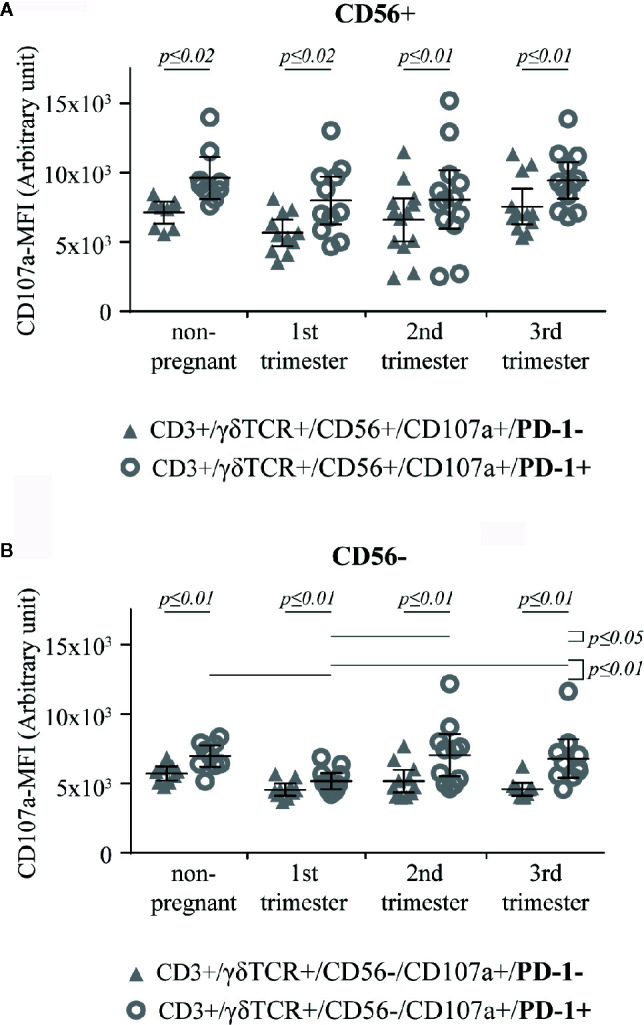
CD107a-MFI in respect of PD-1 positivity in peripheral blood CD56+ **(A)** or CD56− **(B)** γδT cells (CD3+/γδTCR+) in non-pregnant (n = 9) and pregnant [1^st^ (n = 10), 2^nd^ (n = 10), and 3^rd^ (n = 13) trimester] women. The means are depicted as a horizontal line with the 95% confidence interval represented by whiskers. The individual data points are shown as triangles (CD3+/γδTCR+/CD56+/CD107a+/PD-1− or CD3+/γδTCR+/CD56−/CD107a+/PD-1−, respectively) or circles (CD3+/γδTCR+/CD56+/CD107a+/PD-1+ or CD3+/γδTCR+/CD56−/CD107a+/PD-1+, respectively). Statistical analysis was performed by using the paired-samples-t-test to compare data between the PD-1+ and PD-1− γδT cell population and the unpaired-samples-t-test to compare within the same population but at different timepoints. Significant differences are depicted by a horizontal line above the respective data sets.

To determine, if PD-1 expression is related to the intensity of the cytotoxic potential, we finally analyzed the CD107a-MFI on PD-1+ versus PD-1− CD56+/CD107a+ and CD56–/CD107a+ γδT cells, respectively. Here, the CD107a-MFI-value of CD56+/CD107a+/PD-1+ γδT cells was significantly higher in all groups (non-pregnant, 1^st^ trimester: *p* ≤ 0.02; 2^nd^ trimester, 3^rd^ trimester: *p* ≤ 0.01) (**Figure 6A**). This correlation was also significant in CD56−/CD107a+/PD1+ γδT cells (all groups: *p* ≤ 0.01), where among the PD-1+ cells the CD107a-MFI was significantly lower in the 1^st^ trimester compared to all other groups (non-pregnant, 3^rd^ trimester: *p* ≤ 0.01; 2^nd^ trimester: *p* ≤ 0.05) (**Figure 6B**). After statistical comparison of the corresponding data of **Figures 6A, B**, we found a significant higher CD107a-MFI values among CD56+ γδT cells in all groups (all *p* ≤ 0.01).

## Discussion

While αβT cells are traditionally divided into CD4+ and CD8+ T cells, γδT cells are mainly classified based on their Vδ-chain-usage into circulating Vδ2+ and resident Vδ1+ cells. Although, there is a number of reports describing CD8 and/or CD4 positive γδT subtypes or clones (15, 48–50), γδT cells are mainly considered to be double negative and therefore these classical phenotype markers are rarely used in γδT cell research. According to our phenotypic characterization of peripheral blood γδT cells from pregnant and non-pregnant women, it is important to note that γδT cells can express CD4 and CD8. However, we did not find any notable shifts in the rate of CD4 or CD8 expressing cells in the course of pregnancy.

During human pregnancy, a main interface where circulating immune cells can encounter fetal antigens is the syncytiotrophoblast, which covers the embryonic villi of the placenta and is bathed by the maternal blood in the intervillous space. The main characteristic of the syncytiotrophoblast is the lack of HLA Class I and Class II molecule expression (51). Here, γδT and NK cells - as they do not underlie MHC-restriction - are of particular interest. Gamma/delta T cells express several typical NK cell associated receptors, such as NK cell phenotype marker CD56 (52), activating [e.g. NKG2D (53) or NKp44 (54)] or inhibitory NK cell receptors [CD94/NKG2A (55) or p58 (56)]. Therefore, γδT cells are often considered to be a bridge between the innate and the adaptive immune system. So far, CD56+ γδT cells and their functions have mainly been investigated in the aspect of anti-tumor activity, as CD56+ γδT cells show much stronger cytotoxicity following stimulation with IL-2 and IL-12 than CD56− γδT cells (57). It seems that this cytotoxicity is executed *via* the perforin/granzyme pathway and is mainly γδTCR/NKG2D dependent. However, CD56+ γδT cells are resistant to Fas ligand-mediated apoptosis (21). Interestingly, according to our results, half of all CD3+/CD56+ cells were γδTCR+ regardless of existence or stage of pregnancy. In our view, this is a highly important information for all fellow researchers investigating CD56+ T cells. Under physiological circumstances, one to ten percent of peripheral blood T cells are γδT cells (58), which might have led to some negligence of this highly differing subset in T cell research. Due to this high rate of γδT cells among CD3+/CD56+ lymphocytes, γδT cells must be considered in any further “NKT-like” cell research as well. Hereafter, the focus of this study was narrowed to this poorly reviewed T cell subpopulation, CD56+ γδT cells.

To date, the precise role of CD56+ γδT cells in pregnancy has not been described, therefore we aimed to investigate this topic. In our study CD56+ γδT cells were much more common during the 2^nd^ and 3^rd^ trimester, suggesting that they could be especially important in the second half of pregnancy. Regarding the expression of classical T cell phenotype markers CD4 and CD8, the majority of CD56+ γδT cells were double negative, a third of them were CD8+ and just a small percent expressed CD4. Therefore, we assume, that the function of CD56+ γδT lymphocytes could be more cytotoxic than regulatory. However, since the rate of CD4+ cells among CD56+ γδT cells was higher in the 1^st^ and 3^rd^ trimester, these cells might play a role at those specific time points. Further investigation with much higher cell counts are necessary to determine the function of this rare CD4+/CD56+ γδT subpopulation. Compared to CD56− γδT cells, CD56+ γδT cells show a smaller rate of CD4+ cells, while the prevalence of CD8+ cells was higher in CD56+ γδT subset. This relation was prominent in non-pregnant women as well as during pregnancy, with the exception of the 3^rd^ trimester, where both γδT subsets had a similar low CD8-expression.

The CD4/CD8 ratio of CD3+ cells is used as a clinical indicator for the health or functionality of the immune system, whereas a ratio of 1.5 to 2.5 is regarded as normal (59–61). Interestingly and in opposite to CD56+ γδT cells, where we found an inverted CD4/CD8 ratio, CD56− γδT cells’ CD4/CD8 ratio was almost in normal range. Thus, CD56− γδT cells express rather CD4 than CD8. Mincheva–Nilsson described that decidual CD56− γδT cells show an enhanced IL-10 and TGF-β transcription during healthy human pregnancy compared with CD56+ ones, suggesting the immunoregulatory potential of the CD56− γδT cell population (12). It is possible that the CD4+ CD56− γδT cells described in the present study could produce IL-10 and TGF-β.

Besides the regulatory aspect of γδT cells, their cytotoxic potential and its regulation could also be essential for heathy pregnancy. A basic level of cytotoxicity seems necessary for defense against pathogens and tissue remodeling (62). On the contrary, cytotoxicity against the fetus must be prevented. Here we show that the rate of potentially cytotoxic cells is consistently higher among CD56+ γδT cells compared to CD56− γδT but did not differ within the studied non-pregnant or pregnant groups. This general connection of cytotoxic potential and CD56 expression has already been described for CD8+ αβT cells (63) and for anti-tumor γδT cells (21). Interestingly, the potential cytotoxic power (indicated by the CD107a-MFI) of cytotoxic CD56+ γδT cells was low during pregnancy, although it was increasing from the 1^st^ to the 3^rd^ trimester. This finding suggests a weaker cytotoxic burst of CD56+ γδT cells during pregnancy, which might promote fetal survival. This is in line with the previously conducted research in reproductive immunology, that the majority of pregnancy is dominated by a Th2-mediated immune tolerance, which is replaced by a pro-inflammatory period shortly before term (24).

During pregnancy, fetal HLA-G seems to be a major regulator of resident cytotoxic T cells and NK cells (64, 65). However, as HLA-G is expressed by the extravillous cytotrophoblast but not by the syncytiotrophoblast (51), the regulation of circulating cytotoxic cells must be mediated *via* different mechanisms. During the 2^nd^ and 3^rd^ trimester, when the rate of peripheral CD56+ γδT cells is higher in the maternal blood, PD-L1 is also strongly expressed on fetal cyto- and syncytiotrophoblast in the placenta, suggesting a possible association between those (38, 40). With the progression of pregnancy, the maternal placental blood flow increases up to 600 ml/min and the surface area of the syncytiotrophoblast becomes larger (51). At this growing feto-maternal interface, where circulating maternal CD56+ γδT cells can encounter fetal antigens, inhibition *via* PD-1–PD-L1 interactions might be an important control mechanism. Additionally, since PD-L1+ fetal trophoblast cells could leave the placental structure and enter the maternal blood stream (51), these circulating and potentially immunoreactive fetal cells could trigger further effects in the maternal immune system. Altered immunological parameters, which can be detected in the peripheral blood are important in clinical practice, as blood draws are easy, safe and part of standard pregnancy care protocols anyway. To explore the possibility of systemic PD-1-mediated inhibition, we investigated the expression of PD-1 on peripheral CD56+ γδT cells. The rate of PD-1+ cells was generally higher among CD56+ γδT cells compared to CD56− γδT cells. This could imply that CD56+ γδT cells underlie a stronger PD-1-mediated regulation. The prevalence of PD-1+ cells among CD56+ γδT cells is higher in the 1^st^ trimester compared to the non-pregnant group but decreases by the 2^nd^ trimester, which disfavors PD-1 as a major regulator of CD56+ γδT cells at the feto-maternal interface in the 2^nd^ half of pregnancy. For a better understanding of the influence of PD-1 on the cytotoxicity of CD56+ γδT cells during pregnancy, we explored the co-expression of PD-1 and CD107a. This analysis revealed that the previously described 1^st^ trimester increase of PD-1+ cells, however, is only present in PD-1+/CD107a− cells, whereas PD-1+/CD107a+ cells show a decrease in pregnancy (2^nd^ trimester) compared to non-pregnant samples. As previously described, the PD-1 expression is higher in CD56+ than in CD56− γδT cells, and the significance of this difference seems to originate from the CD107a+ subgroup, as the PD-1 co-expression in the CD107a– subgroup is comparable in CD56+ and CD56− cells. This means that whereas the PD-1+/CD107a+ population strongly correlates with CD56 expression, the PD1+/CD107a− phenotype is not dependent on it. Accordingly, we hypothesized that PD-1-mediated regulation should be of higher importance in CD56+ cytotoxic γδT cells. When analyzing the expression of PD-1 within potentially cytotoxic CD56+ γδT cells, just about a quarter of these cells were PD1+ and the prevalence of PD-1+ cells decreased in the 1^st^ trimester and stayed low during pregnancy. On the other hand, cytotoxic CD56– γδT cells showed a peak in PD1 expression only in the 1^st^ trimester. These results suggest, that the PD1 receptor alone might not be able to ensure the entire control of cytotoxicity of peripheral γδT cells during pregnancy. However, when thinking about cytotoxicity, beside the rate of potentially cytotoxic cells, the strength of the potential cytotoxicity (indicated by the CD107a-MFI) of these cells could be more informative. Here, PD-1+/CD107a+ CD56+ γδT cells show a more powerful cytotoxic potential than PD1−/CD107a+ ones during all stages of human pregnancy. This indicates that a PD-1–PD-L1 receptor-ligand interaction could result in an effective inhibition of the threatened high cytotoxicity caused by CD56+ γδT cells. Nevertheless, since a considerable proportion of CD56+ γδT cells are PD1–, it could be supposed that there must be additional mechanisms controlling potential cellular aggression of cytotoxic CD56+ γδT cells. Typical cytotoxicity-inhibiting receptors are NK receptors, like KIR and NKG2A, have HLA molecules as ligands (66, 67), therefore they are possibly not implicated in this regulation. NKG2D, although an activating receptor, could be an appropriate candidate in pregnancy, since the results of Hedlund et al. show that NKG2D could inhibit cytotoxic activity of peripheral blood mononuclear cells after binding syncytiotrophoblast-derived circulating exosomes containing soluble NKG2D-ligands (68).

Nevertheless, pregnancy is a highly dynamic physiological situation, where a higher number of sampling timepoints, especially in the 1^st^ trimester would draw clearer picture. However, studies with a thorough surveillance of immune changes in the course of healthy human pregnancy are rare and as the majority of healthy human pregnancies are not detected before gestation week 5, the extremely interesting phase of implantation and placentation is hard to study.

In this study we demonstrated that half of CD3+/CD56+ lymphocytes showed a γδTCR-phenotype, which was also prevalent over the course of pregnancy. Due to the unique physiology of γδT cells, this observation should be taken in consideration in any further research regarding “NKT-like” cells. Additionally, we demonstrated again the CD4+ and CD8+ phenotype of γδT lymphocytes. The expression of CD4 and CD8 on γδT cells appears to be dependent on the expression of CD56. Although largely double-negative, CD56+ γδT cells express rather CD8+ than CD4+. However, CD4-expressing γδT cells are mainly CD56−. Furthermore, our results indicate that the often-described cytotoxic capacity of γδT cells seems to be mainly represented by the small subset of CD56+ γδT cells, which expands during the 2^nd^ and 3^rd^ trimester of pregnancy. Although the intensity of a potentially cytotoxic burst is low in pregnancy, a small PD-1+/CD107a+ population of CD56+ γδT cells maintain a relatively strong cytotoxic capacity. Evidently, a good immuno-balance is generally important, but for a successful pregnancy it is crucial. Among others, PD-1 expression might prevent potentially harmful overreaction of these highly cytotoxic CD56+ γδT cells at the feto-maternal interface. We hypothesize that conventional CD56− but CD4+ γδT cells, on the other hand, could have an immunoregulatory function, which should be investigated in further studies. Our present findings about γδT cells might help for a deeper understanding of the complex puzzle of immune tolerance in pregnancy.

## Data Availability Statement

The raw data supporting the conclusions of this article will be made available by the authors, without undue reservation.

## Ethics Statement

The studies involving human participants were reviewed and approved by Ethics Committee of the University of Pecs (5643-PTE 2015 and 5643-PTE 2019). The patients/participants provided their written informed consent to participate in this study.

## Author Contributions

The project was conceived by AB and JN and realized by JN. AB provided all critical reagents, experimental support, and critical discussion. PJ ensured the quality of the cytometric data acquisition. The manuscript was written by JN and edited by AB. All authors contributed to the article and approved the submitted version.

## Funding

The present scientific contribution is dedicated to the 650th anniversary of the University of Pécs, Hungary and part of the Hungarian National Laboratory on Reproduction. AB was funded by the University of Pécs Medical School, Hungary (KA-2019-37).

## Conflict of Interest

The authors declare that the research was conducted in the absence of any commercial or financial relationships that could be construed as a potential conflict of interest.
