# Corrigendum: Gamma/Delta T Cells in the Course of Healthy Human Pregnancy: Cytotoxic Potential and the Tendency of CD8 Expression Make CD56+ γδT Cells a Unique Lymphocyte Subset

**DOI:** 10.3389/fimmu.2021.819968

**Published:** 2022-01-14

**Authors:** Jasper Nörenberg, Pál Jaksó, Alíz Barakonyi

**Affiliations:** ^1^ Department of Medical Microbiology and Immunology, Medical School, University of Pécs, Pécs, Hungary; ^2^ Department of Pathology, Medical School, University of Pécs, Pécs, Hungary; ^3^ Janos Szentagothai Research Centre, University of Pécs, Pécs, Hungary

**Keywords:** gamma/delta T cells, human pregnancy, CD4, CD8, CD56, PD-1, cytotoxicity, flow cytometry

In the original article, we neglected to include the funding from **the University of Pécs, Medical School, Hungary (KA-2019-37)** to **Alíz Barakonyi**.

The authors apologize for this error and state that this does not change the scientific conclusions of the article in any way. The original article has been updated.

## Publisher’s Note

All claims expressed in this article are solely those of the authors and do not necessarily represent those of their affiliated organizations, or those of the publisher, the editors and the reviewers. Any product that may be evaluated in this article, or claim that may be made by its manufacturer, is not guaranteed or endorsed by the publisher.

