# Supplementary material for: Gamma/Delta T Cells in the Course of Healthy Human Pregnancy: Cytotoxic Potential and the Tendency of CD8 Expression Make CD56+ γδT Cells a Unique Lymphocyte Subset
Source: Front Immunol. 2021 Feb 2;11:596489. doi: 10.3389/fimmu.2020.596489 (PMC7884463; doi:10.3389/fimmu.2020.596489)
Supplement: Supplementary file 1 [file DataSheet_1.pdf]

Supplementary table 1

Figure 1A (CD3)				
	non-pregnant	pregnant		
		1st trimester	2nd trimester	3rd trimester
γδTCR+/CD56+	2.34 ± 0.35	1.76 ± 0.25	2.12 ± 0.42	1.95 ± 0.23

Figure 1B (γδTCR+)				
	non-pregnant	pregnant		
		1st trimester	2nd trimester	3rd trimester
CD3+/CD56+	48.22 ± 3.42	46.91 ± 3.98	49.31 ± 3.41	51.12 ± 3.38

Figure 1C (CD56+)				
	non-pregnant	pregnant		
		1st trimester	2nd trimester	3rd trimester
γδT cells (CD3+/γδTCR+)	5.19 ± 1.11	4.87 ± 0.47	9.22 ± 0.77	8.39 ± 0.81
non-γδT cells (CD3+/γδTCR-)	3.88 ± 0.6	3.39 ± 0.53	2.14 ± 0.4	2.26 ± 0.39

Figure 2A (CD4-/CD8-)				
	non-pregnant	pregnant		
		1st trimester	2nd trimester	3rd trimester
CD3+/γδTCR+/CD56+	49.27 ± 4.81	44.63 ± 3.78	48.48 ± 4.21	49.23 ± 4.46
CD3+/γδTCR+/CD56-	32.52 ± 3.07	37.01 ± 2.86	35.56 ± 3.45	32.52 ± 2.34

Figure 2B (CD4+/CD8-)				
	non-pregnant	pregnant		
		1st trimester	2nd trimester	3rd trimester
CD3+/γδTCR+/CD56+	8.87 ± 3.05	12.68 ± 1.49	7.88 ± 0.98	11.79 ± 1.75
CD3+/γδTCR+/CD56-	30.35 ± 2.28	28.01 ± 1.83	28.73 ± 2.66	32.69 ± 2.26

Figure 2C (CD4-/CD8+)				
	non-pregnant	pregnant		
		1st trimester	2nd trimester	3rd trimester
CD3+/γδTCR+/CD56+	31.85 ± 3.03	24.88 ± 2.02	28.86 ± 2.88	25.15 ± 3.42
CD3+/γδTCR+/CD56-	24.74 ± 1.47	21.23 ± 1.13	24.08 ± 1.45	24.25 ± 1.45

Figure 3A (CD107a+)				
	non-pregnant	pregnant		
		1st trimester	2nd trimester	3rd trimester
CD3+/γδTCR+/CD56+	46.43 ± 2.82	42.49 ± 2.91	45.86 ± 5.11	44.8 ± 4.23
CD3+/γδTCR+/CD56-	17.23 ± 0.73	21.46 ± 2.13	21.79 ± 2.1	21.26 ± 1.53

Figure 3B (CD107a-MFI)				
	non-pregnant	pregnant		
		1st trimester	2nd trimester	3rd trimester
CD3+/γδTCR+/CD56+/CD107a+	11096 ± 1027	6444 ± 411	7095 ± 497	8181 ± 531
CD3+/γδTCR+/CD56-/CD107a+	5863 ± 219	4593 ± 193	4941 ± 354	4876 ± 228

Supplementary table 1

Figure 4A (PD-1+)				
	non-pregnant	pregnant		
		1st trimester	2nd trimester	3rd trimester
CD3+/γδTCR+/CD56+	15.69 ± 1.35	21.89 ± 1.55	17.59 ± 0.75	16.17 ± 1.06
CD3+/γδTCR+/CD56-	10.55 ± 0.85	15.08 ± 0.69	13.75 ± 0.83	12.59 ± 1.07

Figure 4B (PD-1-MFI)				
	non-pregnant	pregnant		
		1st trimester	2nd trimester	3rd trimester
CD3+/γδTCR+/CD56+/PD-1+	2140 ± 173	2409 ± 149	2388 ± 113	2562 ± 198
CD3+/γδTCR+/CD56-/PD-1+	2257 ± 167	2456 ± 172	2324 ± 113	2435 ± 177

Figure 5B1 (PD-1+/CD107a-)				
	non-pregnant	pregnant		
		1st trimester	2nd trimester	3rd trimester
CD3+/γδTCR+/CD56+	5.52 ± 0.91	10.83 ± 1.21	9.09 ± 1.08	6.88 ± 0.94
CD3+/γδTCR+/CD56-	6.47 ± 0.61	10.39 ± 0.51	9.42 ± 0.84	7.69 ± 0.71

Figure 5B2 (PD-1-/CD107a+)				
	non-pregnant	pregnant		
		1st trimester	2nd trimester	3rd trimester
CD3+/γδTCR+/CD56+	34.04 ± 1.44	31.75 ± 2.52	35.84 ± 4.05	35.35 ± 3.22
CD3+/γδTCR+/CD56-	14.27 ± 0.71	17.48 ± 1.19	16.6 ± 1.21	17.71 ± 1.06

Figure 5B3 (PD-1+/CD107a+)				
	non-pregnant	pregnant		
		1st trimester	2nd trimester	3rd trimester
CD3+/γδTCR+/CD56+	12.86 ± 1.73	11.25 ± 1.19	9.02 ± 1.17	9.64 ± 1.4
CD3+/γδTCR+/CD56-	3.02 ± 0.2	4.23 ± 0.55	4.55 ± 0.51	4.26 ± 0.38

Figure 5C (PD-1+)				
	non-pregnant	pregnant		
		1st trimester	2nd trimester	3rd trimester
CD3+/γδTCR+/CD56+/CD107a+	26.83 ± 1.99	19.47 ± 1.94	20.75 ± 1.65	21.28 ± 1.74
CD3+/γδTCR+/CD56-/CD107a+	17.29 ± 1.26	25.67 ± 1.65	20.22 ± 1.17	19.75 ± 1.35

Figure 6A (CD107a-MFI)				
	non-pregnant	pregnant		
		1st trimester	2nd trimester	3rd trimester
CD3+/γδTCR+/CD56+/CD107a+/PD-1+	9174 ± 875	8036 ± 798	9229 ± 811	9422 ± 688
CD3+/γδTCR+/CD56+/CD107a+/PD-1-	7141 ± 374	6298 ± 329	7530 ± 583	6913 ± 531

Figure 6B (CD107a-MFI)				
	non-pregnant	pregnant		
		1st trimester	2nd trimester	3rd trimester
CD3+/γδTCR+/CD56-/CD107a+/PD-1+	5558 ± 211	4424 ± 193	5018 ± 355	4452 ± 203
CD3+/γδTCR+/CD56-/CD107a+/PD-1-	6777 ± 327	5018 ± 254	6840 ± 665	6608 ± 600
