# Supplementary figures and images for: Gamma/Delta T Cells in the Course of Healthy Human Pregnancy: Cytotoxic Potential and the Tendency of CD8 Expression Make CD56+ γδT Cells a Unique Lymphocyte Subset
Source: Front Immunol. 2021 Feb 2;11:596489. doi: 10.3389/fimmu.2020.596489 (PMC7884463; doi:10.3389/fimmu.2020.596489)

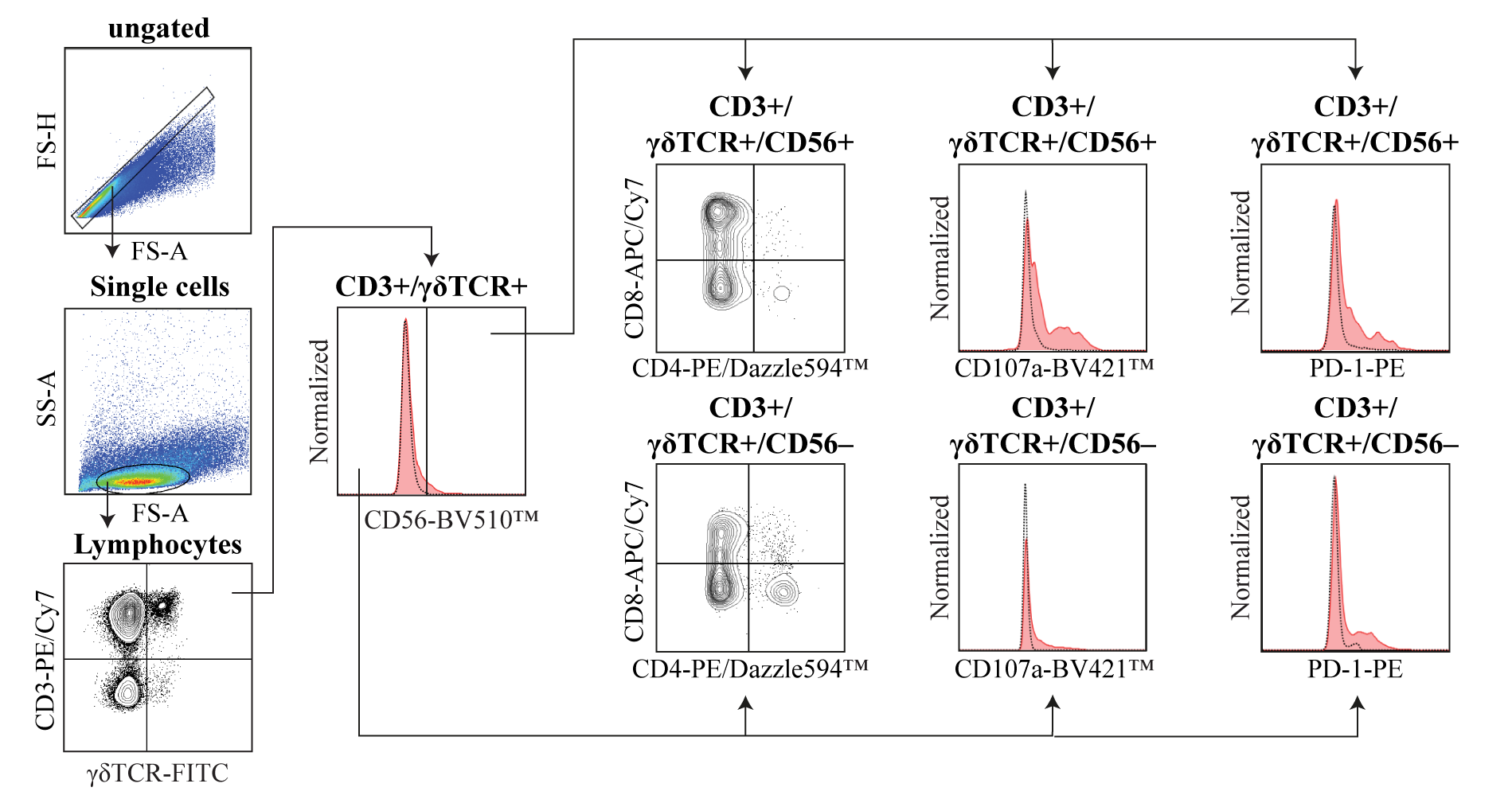

Supplement: Supplementary file 2 [file Image_1.tif]
